# Acute Kidney Injury in Nephrotic Syndrome

**DOI:** 10.3389/fped.2018.00428

**Published:** 2019-01-14

**Authors:** Shina Menon

**Affiliations:** Division of Nephrology, Department of Pediatrics, University of Washington School of Medicine, Seattle Children's Hospital, Seattle, WA, United States

**Keywords:** acute kidney injury, minimal change disease, renal failure, CKD, nephrotic syndrome

## Abstract

Nephrotic syndrome (NS) is one of the commonest kidney diseases seen in childhood and is characterized by a relapsing remitting course. Various complications have been reported in children with NS, including infections, thromboembolism, hypovolemia, and acute kidney injury (AKI). There is often a modest decrease in renal function in patients with active proteinuria due to decreased glomerular permeability that improves when they go into remission. However, more pronounced AKI in NS is multifactorial in origin. It is most often secondary to hypovolemia, nephrotoxic medications, and infections, although other reasons may also be seen. Recent years have seen an increase in the incidence of AKI in NS. There is limited data on the correlation between AKI in pediatric NS and long-term outcomes. A better understanding of this increasingly common condition will help improve patient outcomes.

Nephrotic syndrome (NS) is one of the commonest kidney diseases seen in childhood (1, 2). It is characterized by heavy proteinuria, hypoalbuminemia and anasarca, and generally has a relapsing remitting course. Children with NS are at risk of developing complications like infection, venous thromboembolism (TE), hypovolemia, and acute kidney injury (AKI) (2, 3). While infections and TE can be partly explained by the significant proteinuria seen in NS, AKI in NS is more complicated. It is multifactorial in origin and can be secondary to intravascular volume depletion, acute tubular necrosis, interstitial nephritis, nephrotoxic medications, infections, or renal vein thrombosis (3, 4).

Until recent years most information on the epidemiology of AKI in NS came from case reports. A recent study by Rheault et al. using data from the Healthcare Cost and Utilization Project Kids' Inpatient Database (HCUP-KID) reported a 158% increase in the frequency of NS hospitalizations complicated by AKI between 2000 and 2009 (5). The frequency of hospitalizations secondary to other complications seen with NS like infection and TE remained stable during the same period.

Given the risk of long-term adverse outcomes following AKI in children, it is important to have a better understanding of the problem. This review discusses the recent data on epidemiology, pathophysiology of AKI in NS, and management of this increasingly common complication.

## Epidemiology

AKI is more commonly reported in adults with idiopathic NS. One of the earliest reviews on this topic was by Smith and Hayslett (6). They looked at 79 cases of acute renal failure (ARF) affecting 75 patients with minimal change disease (MCD) reported in literature since 1966. The mean age of patients in their report was 58 years, and ARF was documented at an average of 29 ± 5 days after onset of nephrotic syndrome. Fourteen patients died of uremia or required chronic dialysis, and 3 were lost to follow-up. In the patients who had recovery of renal function (*n* = 58), ARF lasted for 7 weeks. They also noted that while ARF was often attributed to volume depletion, most patients did not improve after correcting volume deficit, and 60% cases showed histopathological changes consistent with acute tubular necrosis (ATN).

A more recent study by Chen et al. looked at 277 patients with idiopathic nephrotic syndrome (7). They defined AKI using the (RIFLE) criteria which includes Risk of renal dysfunction (R), Injury to the kidney (I), Failure of kidney function (F), Loss of kidney function (L), and End-stage kidney disease (E). In their cohort, overall, 95 (34%) patients had AKI with 51 (18%) having RIFLE stage Risk (R); 24 (9%) patients with RIFLE stage Injury (I); and 20 (7%) patients with RIFLE stage Failure (F). Recovery of renal function was seen in 94–100% across the three groups. More severe AKI was associated with longer time to achieve remission and lower rates of complete remission. Severe hypoalbuminemia, older age, and male gender were identified as risk factors of AKI on logistic regression analysis.

In children, lower rates of AKI have traditionally been reported in NS, although this may be in part related to the definitions used. Older studies that describe more severe worsening of renal function or ARF report lower numbers compared to studies that use the newer standardized definitions of AKI. A Polish study reported ARF in 8/1006 (0.8%) children with idiopathic NS (8). They observed that significant hypoalbuminemia, the presence of infection and severe course of nephrotic syndrome were important risk factors for development of ARF. A more recent study based on data from the HCUP-KID for the years 2006 and 2009 showed that 8% of hospitalizations in children with NS had an associated diagnosis of ARF (9).

The rate increases with newer definition of AKI and when more detailed chart review is done. In a single center study from India, Sharma et al. looked at AKI in 355 children with NS (10, 11). They defined AKI using the pediatric modification of RIFLE criteria (p-RIFLE) (11). Overall incidence of AKI was 23.7%; of these 11.2, 7.9, and 4.5% of children had p-RIFLE Stages R, I, and F, respectively. Infection, nephrotoxic medication exposure, and steroid resistant nephrotic syndrome (SRNS) were the main risk factors for development of AKI. The Midwest Pediatric Nephrology Consortium (MWPNC) evaluated children with NS from 17 centers. Using the same p-RIFLE definition, AKI occurred in 58.6%of 336 children and 50.9%of 615 hospitalizations (27.3% in stage R, 17.2% in stage I, and 6.3% in stage F) (3). They reported similar risk factors as the previous study.

Another single center retrospective study on 16 children with SRNS treated with calcineurin inhibitors (CNIs) steroid observed that 13/16 patients (81.3%) had at least one episode of AKI (12). The mean number of AKI episodes was 2.1 ± 1.5 overall.

Table 1 summarizes the recent reports on the epidemiology of AKI in children with NS.

**Table 1:** Summary of recent epidemiological studies in children with Nephrotic Syndrome and Acute Kidney Injury.

**Study, year**	**Definition used**	**Population studied**	**Number of patients with AKI, and rate if available**	**Associated factors**	**Comments**
Kiliś-Pstrusinska et al. (8)	Acute renal failure	Single center, Children with NS aged 6–17.5 years	8/1006 (0.8%)	Hypoalbuminemia Infection	
Rheault et al. (5)	ICD-9-CM diagnostic codes at discharge	Kids' Inpatient Database; ~4,700 hospital discharges per study period	2000: AKI diagnosis in 3.3% of NS discharges 2009: 8.5% of discharges	Older children, females and African-Americans	
Rheault et al. (3)	pRIFLE criteria	17 centers across United States, Age < 18 years	197/336 (58.6%)	Infection: 22% NTMx: 53%	AKI associated with longer LOS
Yaseen et al. (13)	pRIFLE criteria	Single center, Children with NS and AKI aged 2.2–16 years	119 (rate not specified)	Infection: 56% NTMx: 44%	At 3 months, 55% recovered; 41% had CKD; 4% died
Sharma et al. (10)	pRIFLE criteria	< 18 years	84/355 (23.6%)	Infection: 65% NTMx: 35%	Mean time to recovery ranged from 15-28 days based on severity of AKI

*ICD-9-CM, International Classification of Diseases, Ninth Revision, Clinical Modification; NS, Nephrotic Syndrome; pRIFLE, pediatric modification of the Risk of renal dysfunction, Injury to the kidney, Failure of kidney function, Loss of kidney function, and End-stage kidney disease (RIFLE) criteria; NTMx, nephrotoxic medications; AKI, Acute Kidney Injury; CKD, Chronic Kidney Disease*.

## Pathophysiology of AKI

Some degree of renal dysfunction has been reported in children with NS historically. The initial reports described 10–20% patients having elevated blood urea at presentation (14, 15). Later the International Study of Kidney Disease in Children reported that 32% children with minimal change disease (MCD) and 40% of those with focal segmental glomerulosclerosis (FSGS) had serum creatinine levels > 95th percentile of age-matched controls (16). There is a caveat to using creatinine in children with NS. The tubular secretion of creatinine is greatly increased in the presence of heavy proteinuria which can lead to overestimation of the glomerular filtration rate (GFR) significantly (17). This is in addition to the other limitations of using creatinine for the diagnosis of AKI. There is often a delay in the rise of creatinine following injury and it can be diluted in the presence of fluid overload leading to an overestimation of kidney function (18).

The renal dysfunction in NS is generally reversible. Bohlin reported impaired glomerular filtration rate (GFR) by inulin clearance at onset of nephrotic syndrome in six out of 13 children (19). The clinical course of these children did not differ from that of the others over a follow up of 1–7 years, and none had a decreased GFR later in the course of the disease. This reversible renal dysfunction is partly mediated by the podocyte foot process fusion (20). One of the initial studies in this domain by Berg and Bohlin looked at renal hemodynamics in 16 children with biopsy-verified MCD (21). They studied inulin and para-amino hippuric acid (PAH) clearance during proteinuric and non-proteinuric phase of NS and noted that GFR and filtration fraction (FF) were significantly decreased during proteinuric phase compared to controls, while PAH clearance was normal. The same patients, while in remission and in the absence of proteinuria, GFR and FF increased significantly to normal and above-normal values respectively, and PAH clearance remained unchanged. They concluded that normal PAH clearance in the presence of proteinuria ruled out hypovolemia as the cause of reduced GFR, and postulated that the low GFR was likely due to reduced glomerular permeability and/or a reduced filtering surface area. Later studies showed that fusion of foot processes leads to a decrease in the filtration slit frequency (20). This reduces the glomerular capillary wall permeability to water and small solutes like creatinine. The reduction in hydraulic permeability due to decrease in filtration slit frequency was later shown in other studies as well (22). The reduction in glomerular permeability contributes to the reduced GFR in most patients with NS (4).

The traditional “underfill” hypothesis and reduced effective circulating blood volume is no longer believed to be responsible for reduced GFR (4). While most of these patients have low plasma oncotic pressure, there is also increased interstitial pressure along with increased lymphatic flow that maintains a normal blood volume (4, 23). Numerous studies have now shown that in the absence of prolonged diuretic use, most patients with NS do not have reduced circulating blood volume (24–26).

Similar to the underfill hypothesis, other postulated mechanisms for AKI in NS have not withstood the test of time. One such mechanism was impaired filtration from high intratubular pressure secondary caused by protein casts (27). Koomans suggested that at the onset of relapse, heavy glomerular albumin leakage could lead to tubular obstruction. This was also reported in two patients with nephrotic syndrome who rapidly progressed to renal failure (28). The renal histology in these patients showed numerous large protein casts in dilated cortical tubules along with podocyte swelling. The casts and the protein precipitate in the Bowman's capsule reportedly contained varying proportions of albumin and globulin but no Tamm-Horsfall protein and the tubules showed changes from compression by the casts (28). Another theory, “nephrosarca” suggested that interstitial edema in NS induced tubular collapse (29). Ischemic tubular injury and tubular necrosis has been reported in certain series but the underlying mechanism remains unclear (30, 31).

Fujigaki and colleagues looked at tubular cell changes in 37 adult patients with MCD (32). Thirteen patients (35.1%) had AKI by Kidney Disease: Improving Global Outcomes (KDIGO) Clinical Practice Guidelines for AKI criteria at the time of kidney biopsy (33). Both groups were similar with respect to their age, history of hypertension, diuretics use, proteinuria, and serum albumin. While markers of tubular injury (urinary N-acetyl-β-D-glucosaminidase and urinary alpha1-microglobulin) were increased in both groups, they were significantly higher in patient with AKI. They also looked at the immunohistochemical expression of vimentin as a marker of tubular injury and saw that vimentin-positive tubules were high in both groups but the vimentin-positive tubular area per interstitial area was significantly increased in the AKI group. They concluded that proximal tubular injuries were seen in MCD patients without renal dysfunction and were more severe in the presence of renal dysfunction and AKI.

A relatively recent study postulated that adults with AKI in the setting of MCD may have transient circulatory insufficiency at the onset of nephrotic syndrome (7). Chen et al. studied 53 adults with MCD and found that those with AKI had higher blood pressure and a lower serum albumin level than the non-AKI group. Their biopsy revealed more severe foot-process effacement, interstitial edema, and flattening of tubular epithelium. They also found increased expression of endothelin 1 in tubules and glomeruli of patients with AKI than those without. Since endothelin 1 is a vasoconstrictor that acts on the renal vasculature and reduces blood flow and GFR, they hypothesized that there was vasoconstriction and ischemia at the onset of proteinuria and that it was a reversible complication seen in a subset of patients.

## Etiology of AKI in Nephrotic Syndrome

### Hypovolemia

This can be secondary to diarrhea, vomiting, or excessive diuresis, particularly in the setting of hypoalbuminemia.

### Infection

Infections are a common trigger for AKI in NS. A single center study from Pakistan looking at 119 children with NS who developed AKI reported that infections were responsible for 56% of cases (13). This included spontaneous bacterial peritonitis, acute gastroenteritis, sepsis and pneumonia. In the study by Rheault et al. children with infection were twice as likely to develop AKI as children without infection (OR, 2.20; 95% CI, 1.44 to 3.36; *P* < 0.001). According to one review, peritonitis was the triggering event for 4/8 cases of AKI reported (34). The authors hypothesized that peritonitis may worsen the intra- and extrarenal hemodynamics through elevated intraperitoneal production of cytokines like tumor necrosis factor-α and interleukin-6.

### Nephrotoxic Medications

Nephrotoxic exposure is a common cause of AKI in children with NS (3, 12, 13, 35). Rheault and colleagues showed that more than half of all hospitalizations in children with NS frequently were complicated by at least one nephrotoxic medication exposure during the hospital stay, and 20% were exposed to at least two nephrotoxic drugs (3). The common drugs included angiotensin-converting enzyme inhibitors (ACE-Is), calcineurin inhibitors (CNIs), and nephrotoxic antibiotics. Increasing number of nephrotoxic medications, days of exposure, and intensity of exposure are all associated with higher risk for AKI (3).

Other less common causes include renal vein thrombosis, acute interstitial nephritis, or acute tubular injury (3, 4, 36). Rarely a patient may present with rapidly progressive glomerulonephritis (GN) and nephrotic syndrome. This is not commonly seen in idiopathic NS and is often secondary to systemic lupus erythematosus, membranous nephropathy or membranoproliferative GN. These patients have an active urinary sediment (red cells, granular, and cellular casts) and their biopsy may show crescentic GN. AKI may also be seen in patients with the collapsing form of FSGS, where it is secondary to both glomerular and tubular injury.

## Approach to Diagnosis

Evaluation of children who present with AKI and NS depends on the clinical presentation, severity of AKI, and whether the cause of nephrotic syndrome is known.

Most children, with presumed MCD, need only a detailed history and examination along with basic labs to ascertain the cause of AKI. A very small subset will need a kidney biopsy as part of their initial evaluation. A biopsy is usually warranted if the patient has atypical features that may suggest an alternate diagnosis like crescentic GN (active urinary sediment) or collapsing FSGS. Biopsy can also be considered if the child does not have obvious risk factors for developing AKI and does not respond to standard therapeutic measures (volume resuscitation, discontinuation of nephrotoxic medications, treatment of infection).

In recent years, neutrophil gelatinase-associated lipocalin (NGAL) has emerged as a useful biomarker for early diagnosis of AKI (37). NGAL is a small protein expressed in neutrophils and various epithelia, particularly the distal nephron segments of the kidney and its expression is upregulated in response to renal injury (37). It may, in theory help differentiate between prerenal AKI and intrinsic/structural AKI (3). However, in patients with NS, it has not been used for this particular indication. There are some studies that show elevation in NGAL in patients with steroid resistance or cyclosporine toxicity (38, 39). In future, it is possible that newer biomarkers may be discovered to facilitate early diagnosis and more accurate phenotyping of AKI to improve management.

## Management

Management of AKI is mostly supportive. Mild AKI that may be seen at the onset of NS along with edema and proteinuria often resolves as the patient goes into remission with steroids. Those presenting with intravascular volume depletion, infection, or nephrotoxic drugs respond to appropriate management of those inciting factors. While volume replacement helps those who have intravascular volume depletion, albumin infusions have not been shown to be beneficial for the management of AKI. It is often lost immediately in the urine and improves neither serum albumin nor renal function.

## Outcome

Most patients, particularly those who had mild AKI related to impaired glomerular permeability, recover to normal renal function as they go into remission of their nephrotic syndrome. However, as the frequency of AKI seems to be increasing, along with an increased exposure to nephrotoxic medications, there is growing concern about the short and long term effects of AKI in this patient population (3, 5).

In the report from the MWPNC, 12 children (2%) required dialysis (3). Hospital length of stay was longer in children with AKI and increased with the severity of AKI (3). These children were also more likely to require ICU care. Sharma et al. reported that the mean time to recovery of renal function increased with severity of AKI ranging from 15 ± 2 days for AKI stage R to 28 ± 5 days for AKI stage F (10).

Children with NS, particularly those who are steroid resistant and require treatment with CNIs are at risk of developing progressive CKD (3, 12). In recent years, there has been increasing awareness of the AKI to CKD continuum (40, 41). There is however limited data on the link between AKI in NS and progression to CKD.

In a short term outcome analysis following AKI, Yaseen et al. reported recovery of renal function at 3 months in 65/119 children (56%) (13). Of the remaining, 5 patients died and 49 (41.2%) developed varying degrees of CKD. This included 17 (14.3%) patients with CKD stage 2, 18 (15.1%) CKD stage 3, 10 (8.4%) to CKD stage 4, and 4 (3.4%) with CKD stage 5/ESRD. The factors associated with the progression of AKI to CKD in this study were having SRNS, FSGS on histopathology, cyclosporine use and AKI secondary to drug toxicity.

Beins and Dell reported that creatinine returned to baseline in 71% of AKI episodes, while the remaining adopted a new baseline (12). In their study, 6/16 patients (37.5%) developed end stage renal disease (ESRD) during a mean follow up of 6.6 years. The mean number of AKI episodes per patient year of follow-up was significantly associated with the development of ESRD. The estimated GFR was inversely associated with a higher mean number of AKI episodes.

## Conclusion

A drop in GFR from decreased glomerular permeability is not uncommon in NS, and may not have long term implications. However, there seems to be an increase in the rates of AKI in recent years. Given the fairly high prevalence of NS in childhood and the seemingly increasing rates of AKI, one needs to be able to recognize and manage AKI in a timely manner to prevent complications in the future. Adverse outcomes have been reported in the long term following AKI from other causes. Additional studies are needed to determine correlations between AKI in pediatric NS and long-term outcomes.

## Author Contributions

The author confirms being the sole contributor of this work and has approved it for publication.

### Conflict of Interest Statement

The author declares that the research was conducted in the absence of any commercial or financial relationships that could be construed as a potential conflict of interest.
